# Supplementary material for: 13C- and 15N-labeling of amyloid-β and inhibitory peptides to study their interaction via nanoscale infrared spectroscopy
Source: Commun Chem. 2023 Aug 3;6:163. doi: 10.1038/s42004-023-00955-w (PMC10400569; doi:10.1038/s42004-023-00955-w)
Supplement: Supplementary file 2 — Supplementary Information [file 42004_2023_955_MOESM2_ESM.pdf]

Supplementary Information

^{13}C- and ^{15}N-labeling of amyloid-β and inhibitory polypeptides to study their interaction via nanoscale infrared spectroscopy

Suman Paul[1,4], Adéla Jeništová[1], Faraz Vosough[1], Elina Berntsson[1,2], Cecilia Mörman[1,5], Jüri Jarvet[1,3], Astrid Gräslund[1], Sebastian K. T. S. Wärmländer[1], Andreas Barth[1*]

[1] Department of Biochemistry and Biophysics, Stockholm University, Stockholm, Sweden
[2] Department of Chemistry and Biotechnology, Tallinn University of Technology, Tallinn, Estonia
[3] National Institute of Chemical Physics and Biophysics, Tallinn, Estonia
[4] present address: attocube systems AG, Haar, Germany
[5] present address: Department of Biosciences and Nutrition, Karolinska Institutet, Huddinge, Sweden

* barth@dbb.su.se

Supplementary Note 1: nano-FTIR spectra averaged over several locations

Fig. S1 shows average nano-FTIR spectra for unlabeled Aβ40 (blue), labeled Aβ40 (red), and unlabeled NCAM1-PrP fibrils (green). The Aβ40 spectra were calculated from the spectra shown in Fig. 1 of the main text.

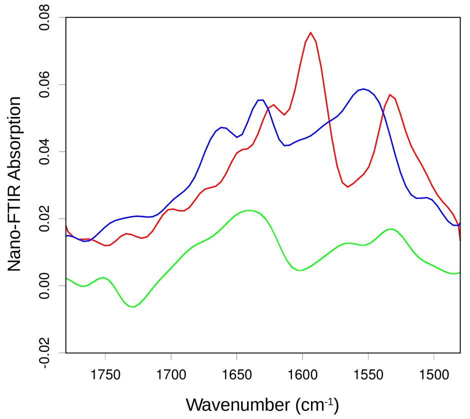

Fig. S1. Average of the Aβ40 spectra shown in Fig. 1 and of NCAM1-PrP spectra. Blue: unlabeled Aβ40 (average of 5 spectra, main bands at 1632 and 1554 cm^{-1}), red: labeled Aβ40 (average of 4 spectra, 1594, 1533 cm^{-1}), green: NCAM1-PrP (average of 18 fibril spectra, 1640, 1533 cm^{-1}). The spectra were not normalized. They are vertically shifted for a clearer presentation.

Supplementary Note 2: Mismatch of uncropped phase images

The phase images at the two wavenumbers did not cover exactly the same area but were slightly misaligned. When this misalignment was not taken into account and the difference image was calculated without cropping, this resulted in features where positive and negative values ran parallel as shown in Fig. S2. The missing of such parallel features was taken as criterion for a correct alignment of the two cropped images.

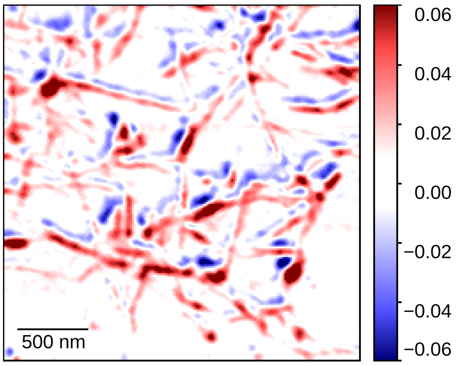

Fig. S2. Difference image ($\Phi_{1587} - \Phi_{1629}$) showing the mismatch of the original phase images at 1587 cm^{-1} and 1629 cm^{-1} for labeled Aβ40.

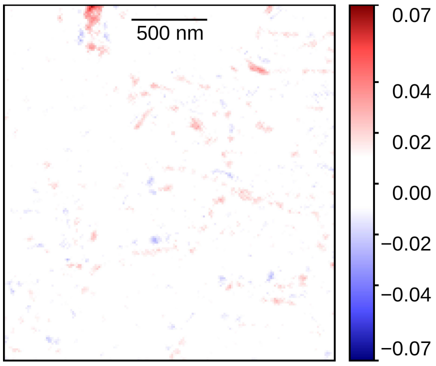

Fig. S3. Difference image (Φ_{1587} − Φ_{1587}) of two subsequent scans of the same area of the Aβ40 sample at 1587 cm^{-1}. The color scale is the same as in Fig. 2 and Fig. S4 for the same sample.

Supplementary Note 3: Level of confidence in difference images

Fig. S3 shows a difference image that was generated from two subsequent scans at 1587 cm^{-1} of the same area of an Aβ40 sample. Ideally, the signals of the two scans should cancel when the difference image is calculated. However, small difference features are observed that partly correlate with structural features shown in Fig. 2. Often, there are parallel blue and red features which indicate an imperfect alignment of the individual images from each scan. This is difficult to avoid because the best alignment often requires cropping one image by a non-integer number of pixels and because the optimum crops are different in different regions of the image. The latter implies that a particular feature of interest can be aligned better than the entire image and therefore can be analyzed with a higher level of confidence.

Nevertheless, the phase differences in Fig. S3 are much smaller than the differences of the phase at 1587 and at 1629 cm^{-1} shown in Fig. 2 (top row, column F) and in Fig. S4 (top left) which both have the same color scale as Fig. S3.

Supplementary Note 4: Difference images and line profiles of Aβ aggregates

The difference images (phase at 1587 cm^{-1} minus phase at 1629 cm^{-1}) shown in the main text are compiled in Figs. S4 and S5 together with phase images at the two individual wavenumbers and with phase profiles along specific lines. Table S1 lists the heights of the aggregates that are encountered by the lines. Fig. S4 shows such results for the control experiments where all material was either labeled or unlabeled. Fig. S5 shows respective results obtained with mixtures of labeled and unlabeled peptides. The figures are discussed in the main text.

Table S1. Aggregate heights for the line profiles shown in Figs. S4 and S5. The heights are listed from left to right in the phase profile plots. To facilitate the correlation of the height with the features in the images, also the distance d in nm from the start of the line is stated as subscript for some of the lines. The first digit is given for heights < 5 nm.

Sample	Figure	Line	Aggregate heights / nm
Aβ40	S4a-d	1	1.8
		2	10, 12, 12, 9
		3	7
		4	4.5, 3.4, 9, 8
Aβ40(L)	S4e-h	1	1.0, 1.9, 13, 7
		2	1.8
		3	5
		4	11, 15, 3.5, 21
		5	1.6, 2.2, 8
Aβ42	S4i-l	1	1.8, 0.5, 3.1, 1.5, 3.4
		2	1.5, 1.8
		3	1.8, 1.7, 4.7
		4	2.3, 3.5
		5	2.5
		6	2.0
		7	3.4
		8	4.4, 1.6
NCAM1-PrP	S4m-p	1	2.5, 2.0, 2.3
		2	2.5_{400}, 2.5_{555}, 2.0_{620}, 2.5_{770}
		3	2.3
		4	3.0_{80}, 1.8_{520}, 2.6_{640}, 3.1_{750}, 1.1_{950}
		5	2.0
		6	3.3
		7	2.6
Aβ40 + Aβ40(L)	S5a-d	1	2.4_{270}, 11_{830}, 4.4_{970}, 32_{1570}, 10_{1970}, 3.2_{2070}
		2	1.2, 1.2, 1.5
		3	11_{350}, 26_{570}, 15_{750}, 11_{1190}, 2.5_{1470}
		4	2.0, 8.0, 1.8, 2.0
		5	3.6
Aβ42 + Aβ40(L)	S5e-h	1	15, 5, 4.4, 4.8
		2	0.3_{120}, 0.5_{230}, 1.9_{370}, 7_{580}, 16_{1240}, 12_{1750}
		3	0.5_{100}, 0.5_{230}, 14_{540}
		4	2.6_{200}, 0.8_{460}, 4.3_{660}, 4.6_{1130}, 0.7_{1330}, 1.2_{1530}, 0.9_{1880}, 13_{2340}, 4.0_{2540}, 9_{2780}
		5	11_{570}, 15_{730}, 16_{860}
		6	4.0
		7	4.2, 1.7, 0.9
		8	1.1, 3.5
		9	11_{390}, 8_{480}
		10	1.2
		11	2.1
		12	1.5, 1.3
		13	0.6, 0.5, 2.9
Ncam1-PrP + Aβ40(L) Location 1	S5i-l	1	6, 14, 4.8, 8
		2	25_{430}, 9_{550}, 13_{690}, 8_{1730}
		3	2.0_{600}, 2.8_{940}, 12_{1430}, 8_{1570}, 7_{1890}, 6_{2010}
		4	9, 3.4
		5	10, 6
Ncam1-PrP + Aβ40(L) Location 2	S5m-p	1	1.8
		2	6
		3	20_{310}, 2.0_{410}, 3.0_{610}, 13_{870}, 11_{1030}, 3.0_{1250}
		4	6_{150}, 4.6_{250}, 3.0_{290}, 29_{740}, 10_{890}, 14_{1200}
		5	6, 6
		6	13

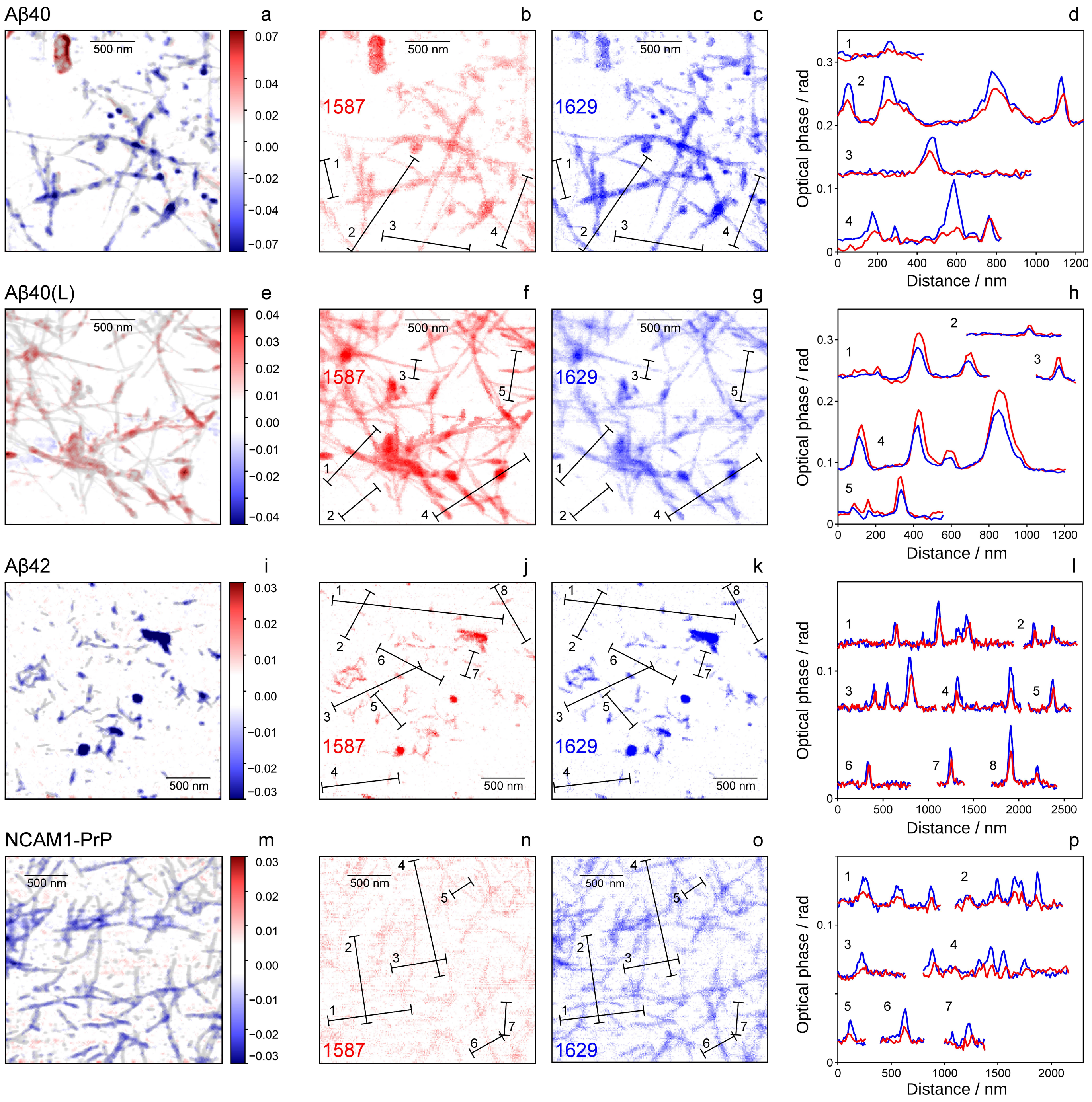

Fig. S4. Nanoscale infrared imaging of control samples. a-d: unlabeled Aβ40; e-h: ^{13}C-,^{15}N-labeled Aβ40; i-l: unlabeled Aβ42; m-p: unlabeled NCAM1-PrP. a, e, i, m: difference image of the optical phase at 1587 cm^{-1} (characteristic for labeled peptide) minus the optical phase at 1629 cm^{-1} (characteristic for unlabeled peptide) overlayed with a height image. Positive values in the difference image are coded in red and indicate more absorption at 1587 cm^{-1} than at 1629 cm^{-1}, which is characteristic of the labeled peptide. Negative values are shown in blue and reveal more absorption at 1629 cm^{-1}, which is characteristic of the unlabeled peptide. Phase differences close to zero are shown in white. The scale of the phase difference image is symmetrical around zero, *i.e.* the absolute values of the maximum and minimum scale values are the same. The overlayed semi-transparent height image indicates the height in a gray scale. Gray regions without a blue or red color tone indicate peptide aggregates where the phase difference is close to zero. b, f, j, n: optical phase at 1587 cm^{-1}; c, g, k, o: optical phase at 1629 cm^{-1}. The color scale of both corresponding phase images for each sample extends over the same range. d, h, l, p: profiles of the optical phase along lines indicated in the phase images. The line profiles were drawn between matching points in the two corresponding phase images from left to right and with 10 pixel thickness (pixel size: 12.5 nm x 12.5 nm). The line numbers are close to the start of the line. Red: line profile at 1587 cm^{-1}, blue: line profile at 1629 cm^{-1}.

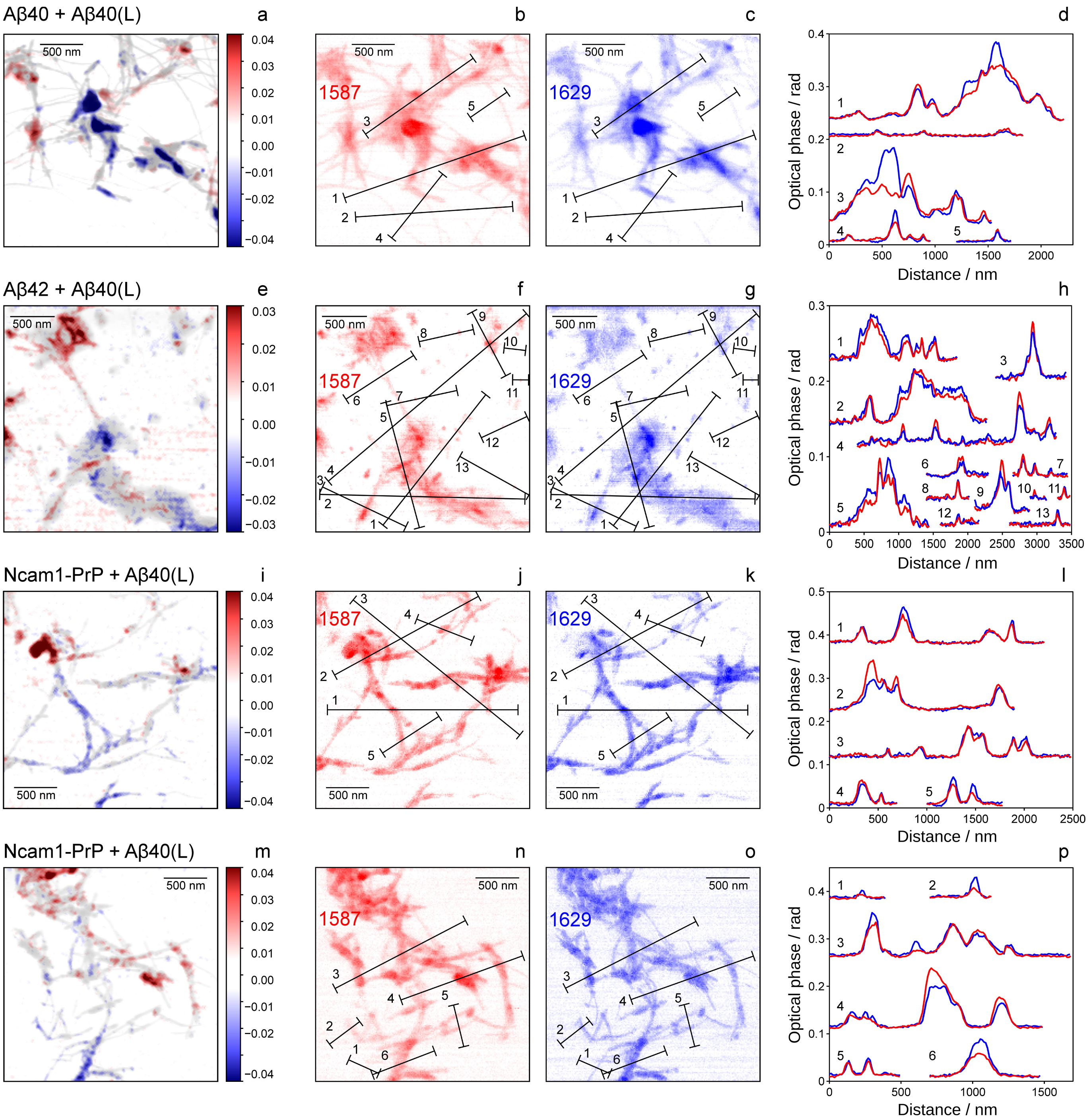

Fig. S5. Nanoscale infrared imaging of mixtures of unlabeled peptides with labeled Aβ40.

a-d: 1:1 mixture of labeled and unlabeled Aβ40; e-h: 1:1 mixture of unlabeled Aβ42 and labeled Aβ40; i-l: 1:1 mixture of unlabeled NCAM1-PrP and labeled Aβ40, location 1; m-p: 1:1 mixture of unlabeled NCAM1-PrP and labeled Aβ40, location 2. a, e, i, m: difference image of the optical phase at 1587 cm^{-1} (characteristic for labeled peptide) minus the optical phase at 1629 cm^{-1} (characteristic for unlabeled peptide) overlayed with a height image. Positive values in the difference image are coded in red and indicate more absorption at 1587 cm^{-1} than at 1629 cm^{-1}, which is characteristic of the labeled peptide. Negative values are shown in blue and reveal more absorption at 1629 cm^{-1}, which is characteristic of the unlabeled peptide. Phase differences close to zero are shown in white. The scale of the phase difference image is symmetrical around zero, *i.e.* the absolute values of the maximum and minimum scale values are the same. The overlayed semi-transparent height image indicates the height in a gray scale. Gray regions without a blue or red color tone indicate peptide aggregates where the phase difference is close to zero. b, f, j, n: optical phase at 1587 cm^{-1}; c, g, k, o: optical phase at 1629 cm^{-1}. The color scale of both corresponding phase images for each sample extends over the same range. d, h, l, p: profiles of the optical phase along lines indicated in the phase images. The line profiles were drawn between matching points in the two corresponding phase images from left to right and with 10 pixel thickness (pixel size: 12.5 nm x 12.5 nm). The line numbers are close to the start of the line. Red: line profile at 1587 cm^{-1}, blue: line profile at 1629 cm^{-1}.
